# Embedded Droplet Velocity Sensor for Lab-on-PCB Systems: A Case Study on Velocity and Monodispersity in Flow-Focusing Systems

**DOI:** 10.3390/mi17101178

**Published:** 2026-10-11

**Authors:** Daniel Solano, Jair Gonzalez-Pina, Antonio Luque, Sergio Camacho-Leon

**Affiliations:** 1 Tecnologico de Monterrey, School of Engineering and Sciences, Ave. Eugenio Garza Sada 2501 Sur, Col. Tecnologico, Monterrey 64700, Nuevo Leon, Mexico; danielsolano@exatec.tec.mx; 2 Global Tech Department, Ansys (Part of Synopsys), Canonsburg, PA 15317, USA; 3 Electronics Engineering Department, Universidad de Sevilla, 41092 Sevilla, Spain

**Keywords:** flow-focusing microfluidics, embedded sensing, real-time monitoring, droplet velocity measurement, monodispersity, model-based systems engineering, optoelectronic detection, Lab-on-PCB

## Abstract

Droplet velocity measurement in miniaturized Lab-on-PCB (LoPCB) platforms is essential for monitoring flow regime stability, monodispersity, and downstream processing in applications such as nanoparticle synthesis and clinical diagnostics, yet conventional optical techniques require bulky external instrumentation unsuitable for autonomous operation. This work presents a fully embedded opto-electrical velocity sensing system that performs sensing, signal conditioning, and data processing on-board, without requiring external instrumentation. The sensor architecture was designed following a Model-Based Systems Engineering (MBSE) workflow and validated through simulation prior to microcontroller deployment. The maximum detectable velocity, set by photoresistor response dynamics, increases linearly with droplet length. Experimental validation was performed in a 1.4 mm flow-focusing geometry using water-in-oil emulsions across flow-rate ratios (ϕ) from 0.5 to 2.0 under a fixed continuous-phase flow rate ($Q_{c}$). The system achieved an average deviation of 5.32% from optical reference values across 195 droplets, with per-condition deviations ranging from 0.74% to 12.07% and below 6.4%, excluding the highest-dispersion condition. Embedded analysis of 797 droplets revealed a linear trend between velocity and droplet length ($R^{2}=0.993$) and a power-law relationship between velocity and monodispersity ($R^{2}=0.912$) over the 3.7–7.4 mm/s range. These results demonstrate suitability for autonomous, real-time droplet characterization in Lab-on-PCB platforms.

## 1. Introduction

Droplet microfluidic systems have emerged as the gold standard for developing miniaturized analytical platforms such as Lab-on-Chip (LoC) devices, owing to their ability to precisely generate, manipulate, and process small liquid volumes dispersed in a continuous phase [1]. This technology enables a wide range of applications, including single-cell analysis [2,3], nanoparticle synthesis [4], polymerase chain reaction (PCR) [5], drug screening [6], and enzyme-catalyzed reactions [7]. In these applications, accurate control and characterization of key parameters—such as flow rate, droplet velocity, droplet length, and monodispersity—are essential for achieving reliable and reproducible operation.

Among these parameters, droplet velocity plays a crucial role because it directly affects flow regime stability, monodispersity, mixing efficiency, and downstream processing. However, velocity measurements in droplet microfluidic devices are typically performed off-chip, requiring external instrumentation such as high-speed cameras, combined with image-processing algorithms, numerical simulations, or machine learning techniques [8,9,10,11]. While effective, these approaches hinder the portability of the final system and significantly increase its cost and complexity. This limitation becomes particularly critical for Lab-on-PCB (LoPCB) platforms, which aim to deliver low-cost, scalable solutions compatible with standard printed circuit board (PCB) fabrication technologies.

To overcome these constraints in LoC/LoPCB devices, recent advances have led to the emergence of embedded microfluidic systems, which integrate sensing, actuation, and processing within a single platform. For instance, Li et al. demonstrated a smartphone-based system for portable liquid handling [12], although external bench microscopy is still required for droplet detection. Similarly, Nguyen et al. presented the integration of point-of-care devices with IoT technology [13], where the incorporation of embedded sensing could further strengthen large-scale clinical validation and real-world deployment robustness. These examples illustrate the growing convergence between microfluidics and Cyber–Physical Systems (CPSs), where physical fluidic operations are augmented through embedded computation and communication networks [14]. Achieving this integration requires that the characterization and sensing of key parameters be performed within the embedded hardware itself, enabling real-time feedback and adaptive control for more robust and autonomous operation.

In this context, the present work introduces an embedded sensing architecture for droplet-based microfluidic systems that combines edge-based detection with embedded velocity estimation using a minimal hardware footprint. In [15], the optoelectronic detection technology based on LDRs was characterized for Lab-on-PCB applications, with signal analysis performed offline in MATLAB (R2024b). Subsequently, ref. [16] introduced modular detection algorithms for droplet-length and monodispersity analysis, although velocity characterization still relied on external video-based methods. The present work addresses these gaps by integrating droplet generation, velocity analysis, and droplet assessment within a single embedded platform. The system is designed following Model-Based Systems Engineering (MBSE) principles to support modular integration and simulation-based verification.

Finally, the performance of the proposed system was validated through an embedded case study investigating the influence of droplet velocity on monodispersity in water-in-oil emulsions using a flow-focusing microfluidic configuration. The analysis of 797 droplets demonstrated an inverse correlation between droplet velocity and monodispersity, thereby confirming the system’s effectiveness in enabling autonomous, real-time characterization of droplet formation dynamics.

## 2. System Design

**Figure 1:**
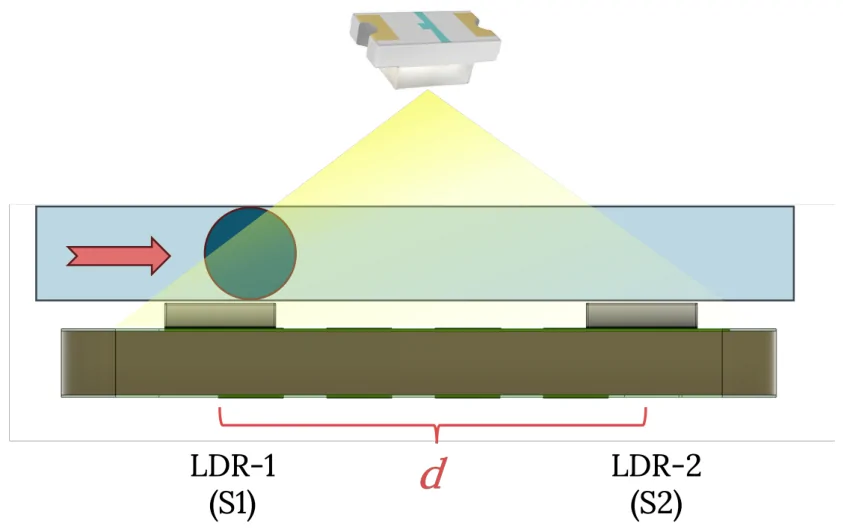
Schematic of the optical velocity sensing setup. A single LED illuminates two photoresistors (S1 and S2) positioned at a fixed distance along the outlet channel. Droplet passage is detected by light interruption at each sensor, and the time between events is used to determine droplet velocity. The red arrow indicates the flow direction, and the blue circle represents a water droplet passing over S1.

The sensor design consists of an array of light-dependent resistors (LDRs) positioned beneath the main channel of the flow-focusing geometry, separated by a known distance (d). To improve light-variation detection, a light-emitting diode (LED) was placed above the channel, as shown in Figure 1. This configuration enables optical droplet detection and provides a simple, non-invasive method to monitor droplet dynamics downstream of the generation region, following the fabrication protocols described in [15,16]. Importantly, this approach performs optical detection externally to the microchannel, leveraging low-cost PCB-mounted components and eliminating the need to integrate electrodes or optical elements within the microfluidic device itself.

The sensing system employs miniature cadmium sulfide (CdS) photoresistors (SG36516) with a measured dark resistance of 16.87 MΩ and illuminated resistance of 6.63 kΩ under nominal LED intensity. Under experimental conditions, the LDR exhibits a rise time (10–90%) of $\tau_{\mathrm{rise}}=9.14\pm 0.33$ ms and fall time of $\tau_{\mathrm{fall}}=36.75\pm 2.76$ ms, measured via oscilloscope characterization.

The light source is a green LED operated at approximately 21 mA via a 150 Ω current-limiting resistor from a 3.3 V supply. The LED broadband emission overlaps well with the CdS spectral sensitivity (500–600 nm range), and stable illumination is maintained without active thermal control due to the low power dissipation (<70 mW).

The fabricated PCB sensing module includes two LDRs, S1 and S2, separated by a center-to-center distance of d=9.88mm, measured from the PCB layout artwork. The embedded system measures the transit time of each droplet between the two sensors, and the droplet velocity *V* is computed according to the definition in Equation (1) where *d* is the fixed distance between sensors S1 and S2, and Δt is the time difference between consecutive detections.

(1) $$V=\frac{d}{\Delta t}$$

The electronics and PCB layout were designed around a Schmitt-trigger comparator circuit to enable high-speed droplet detection and avoid on-board analog-to-digital conversion (ADC) [17]. Two voltage reference values were applied to the inputs of the LM393 comparator [18] to ensure precise transitions between the dispersed phase (water) and continuous phase (oil); the references of S1 and S2 are set independently by digital potentiometers. When either sensor detects the dispersed phase, the comparator output switches to HIGH, and the digital signal is sent to the microcontroller for processing within the embedded system.

**Figure 2:**
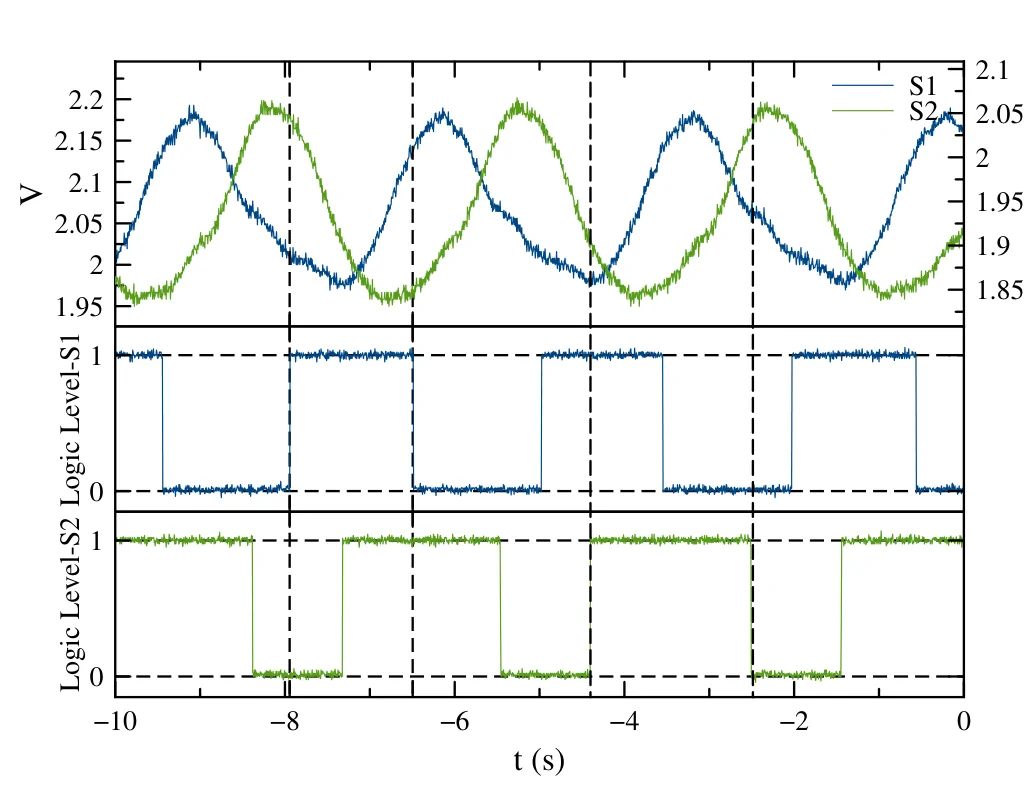
Real-time signals acquired from a 1.4 mm flow-focusing droplet generator. (**Top panel**): analog voltage signals from sensors S1 (blue) and S2 (green). (**Middle panel**): digital comparator output for S1 (blue). (**Bottom panel**): digital comparator output for S2 (green). Rising edges on the digital traces indicate droplet detection events; the time difference Δt between corresponding S1 and S2 edges yields the droplet transit time. Vertical dashed lines mark droplet detections at S1 and S2, and horizontal dashed lines indicate logic levels 0 and 1.

The corresponding signal behavior is illustrated in Figure 2. The top panel shows the analog voltage signals from LDRs S1 (blue) and S2 (green), while the bottom panels display the digital outputs of the Schmitt-trigger comparator: the middle panel (blue trace) corresponds to S1 and the lower panel (green trace) to S2.

### Operational Velocity Limits of the Optical Sensing System

While the electronics impose a theoretical upper bound on measurable velocity—set by the 500 μs software debounce interval (chosen to suppress spurious comparator re-triggering) and the 1.3 μs comparator response time, yielding $V_{\max,\mathrm{elec}}=d/\Delta t_{\min}=19.76$ m/s for d=9.88 mm—the practical velocity limit is governed by the LDR response dynamics.

Since the LDR fall time ($\tau_{\mathrm{fall}}=36.75\pm 2.76$ ms) determines how quickly a passing droplet is detected, the comparator threshold—set at 50% of the voltage swing—is reached after a detection delay of $t_{\mathrm{detect}}=\ln\left(2\right)\;\tau \approx 11.5$ ms, where $\tau =\tau_{\mathrm{fall}}/\;\ln\left(9\right)$ is the underlying RC time constant obtained from the standard 10–90% to time-constant conversion. For reliable detection, a droplet must block each sensor for at least this duration, imposing the temporal constraint, as described in Equation (2), where $L_{\mathrm{drop}}$ is the droplet length in the flow direction.

(2) $$V_{\max}=\frac{L_{\mathrm{drop}}}{t_{\mathrm{detect}}}$$

**Figure 3:**
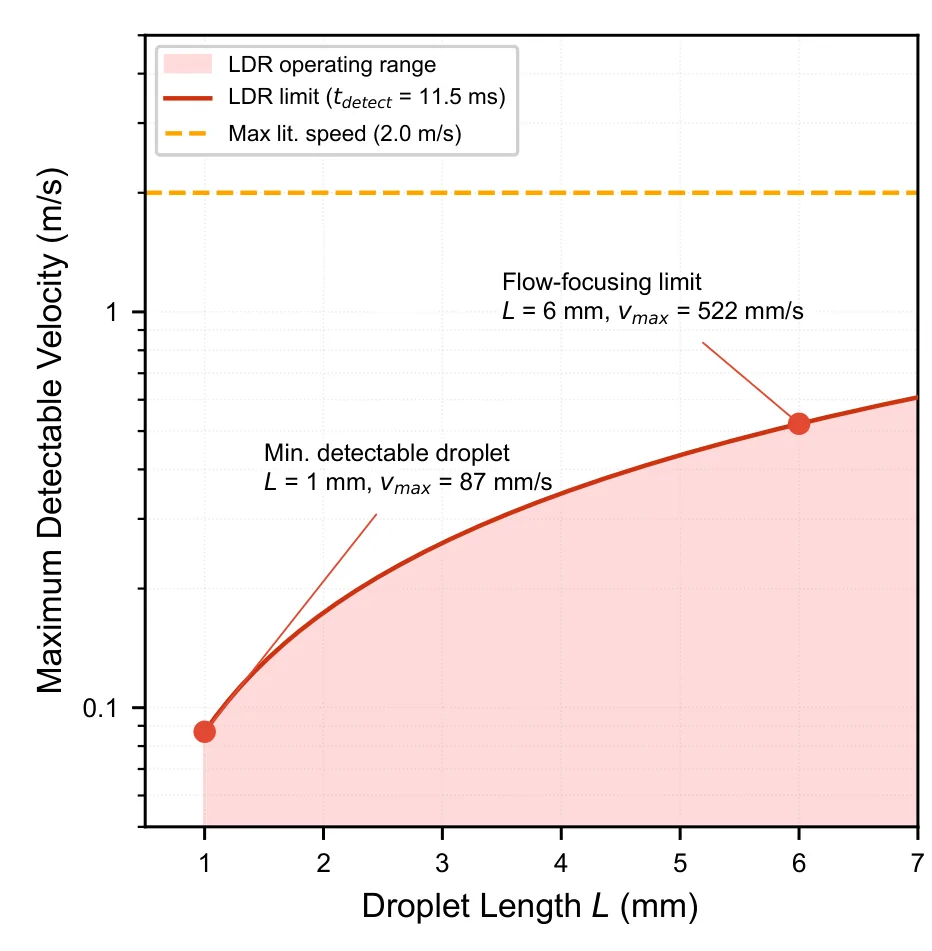
Maximum detectable droplet velocity as a function of droplet length for a detection delay of $t_{\mathrm{detect}}=11.5$ ms. The shaded region represents the velocity–length combinations detectable by the LDR-based sensor. The lower bound corresponds to the minimum detectable droplet length (L=1 mm, $v_{\max}=87$ mm/s), and the upper bound (L=6 mm, $v_{\max}=522$ mm/s, L/W≈4.3) corresponds to the maximum droplet length consistent with squeezing-regime scaling [19].

An additional geometric constraint arises from the LDR active sensing area (2.4 mm × 1.4 mm), whose 1.4 mm height closely matches the 1.4 mm channel width of the flow-focusing device. For effective light blockage, the droplet cross-section must substantially fill the sensing area ($d_{\mathrm{drop}}\geq 1.0$–1.4 mm), which sets the shortest detectable droplet. Beyond this minimum length, the maximum detectable velocity increases linearly with $L_{\mathrm{drop}}$ (Figure 3), and the value of 522 mm/s corresponds to $L_{\mathrm{drop}}=6$ mm, the upper end of the length range in the figure; it is therefore specific to elongated plugs and not a general limit of the sensor.

The upper bound of the droplet length range in Figure 3 (L=6 mm, L/W≈4.3) was determined using the Garstecki scaling law $L/W=1+\alpha \;(Q_{d}/Q_{c})$ [19], where stable plug generation in the squeezing regime has been demonstrated up to L/W≈10 [20]. This confirms that the sensor operating range is well matched to the droplet sizes produced by conventional flow-focusing geometries.

According to the literature, droplet velocities in microfluidic systems typically range from 0.01 to 2 m/s, the latter being the maximum velocity reported in the literature [21,22,23]. The proposed sensor covers the low-to-moderate velocity range characteristic of flow-focusing and T-junction devices operating in the squeezing regime, with a maximum detectable velocity that depends on droplet length (Figure 3).

The LDR response dynamics also set a theoretical upper bound on the droplet rate that each sensor can resolve. Since each droplet must block the sensor for at least $t_{\mathrm{detect}}$ and the LDR must recover within $\tau_{\mathrm{rise}}$ before the next droplet arrives, this bound is approximately $1/(t_{\mathrm{detect}}+\tau_{\mathrm{rise}})\approx 48$ Hz.

Although the velocity range is bounded by the LDR response dynamics, photoresistors offer a high intrinsic sensitivity (2544× ($R_{\mathrm{dark}}/R_{\mathrm{light}}$)), circuit simplicity, and low cost, enabling robust threshold detection with only two passive components per sensing channel. These attributes make the LDR-based architecture particularly suited for affordable, rapid prototyping of modular droplet-based microfluidic sensors in Lab-on-PCB applications. For scenarios demanding higher velocities, droplet rates, or smaller droplets, photodiodes with transimpedance amplifiers remain a viable alternative at increased circuit complexity, fabrication effort, and 3–25× higher cost.

## 3. Embedded System

The embedded implementation of the droplet velocity sensor was developed following a Model-Based Design (MBD) methodology using Ansys SCADE (Safety-Critical Application Development Environment), a model-based development environment for embedded systems [24]. SCADE provides a graphical modeling environment with automatic ANSI C code generation, which was used to implement and verify the embedded logic prior to deployment. The system integrates all signal acquisition, processing, and velocity estimation functionalities within a single embedded unit, enabling fully embedded operation without external computation.

### 3.1. Interruption-Based Velocity Sensor Implementation

The velocity sensor subsystem was modeled as an interruption-driven operator within SCADE, replicating the behavior of the Arduino Zero’s Interrupt Service Routines (ISRs). Comparator outputs from the two light sensors (S1 and S2) were mapped to rising-edge digital interrupts on the ARM Cortex-M0+ microcontroller (SAMD21, 48 MHz; Microchip Technology Inc., Chandler, AZ, USA; Arduino Zero, Arduino S.r.l., Monza, Italy), selected for its compatibility with the SCADE ANSI C code generator, low cost, and open-source ecosystem. Each ISR captures a timestamp using the internal micros() function (1 μs resolution) and sets a detection flag.

**Figure 4:**
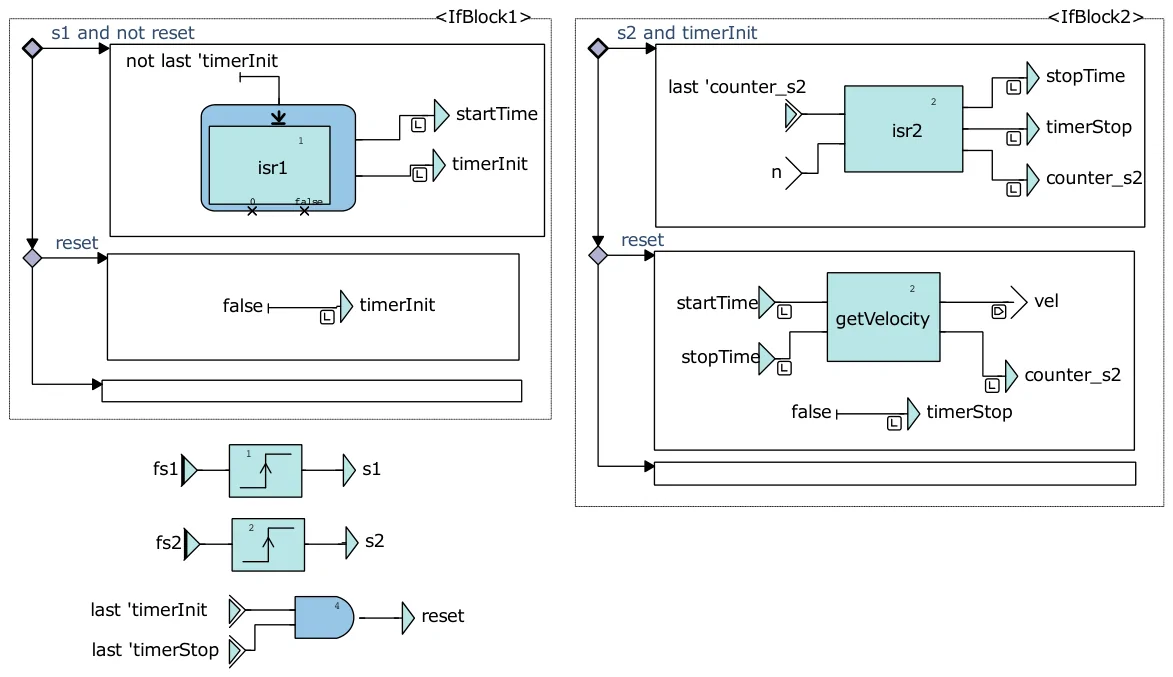
SCADE model for the velocity sensor subsystem. The blocks isr1 and isr2 capture timestamps when the respective interrupt flags fs1 and fs2 are triggered. The getVelocity block calculates droplet speed using the time difference and fixed sensor spacing.

Figure 4 illustrates the SCADE model, which implements an interruption-based architecture wherein fs1 and fs2 are the ISR outputs triggered by comparators S1 and S2. These signals are processed by the isr1 and isr2 operators, which capture the precise detection times using micros(). Once both timestamps are available, the system activates the getVelocity block, which computes the droplet velocity based on Equation (1). This modular design ensures high time resolution and robust synchronization of detection events while adhering to embedded real-time constraints.

This interruption-based approach minimizes CPU usage by eliminating polling and reducing detection latency, an essential feature for accurately measuring high-speed droplets. Once validated through simulation, the whole model was automatically translated into ANSI C code using the SCADE code generator and deployed onto the target microcontroller, ensuring that the implemented binary is behaviorally equivalent to the verified model.

### 3.2. Integration of Droplet Detection and Characterization Modules

In addition to velocity sensing, the embedded system incorporates modular algorithm blocks from our previous work [16], including droplet detection, counting, and monodispersity analysis. These modules were implemented using the same SCADE model-based workflow and integrated as independent components with shared access to detection flags and timestamp buffers. The signal processing algorithms rely on first- and second-order derivative criteria to identify local minima and maxima for robust peak detection.

Furthermore, droplet detection, counting, and length estimation follow the analytical equations defined in [16], enabling consistent characterization across experiments. The model-based design environment facilitated the debugging and integration of these modules with the velocity sensor. The system operates in two sequential modes—velocity measurement and droplet characterization—and the SCADE simulation environment allowed verification of correct mode switching, ensuring proper state transitions between modes prior to code generation. The graphical model representation further simplified the review and validation of the integrated system logic, and the automatically generated ANSI C code provides platform portability, enabling deployment to any C-compatible embedded target without modifying the application logic. As a result, the platform enables fully embedded detection, real-time characterization, and parameter logging within a compact, low-power design.

## 4. Experimental Setup

The experimental validation was conducted using a flow-focusing microfluidic device fabricated in polymethyl methacrylate (PMMA) and bonded using a thermal press technique, as described in [16]. The channel geometry consisted of a square cross-section with a width of 1.4 mm, integrated into the platform shown in Figure 1 and enclosed within the housing previously designed in [16]. The housing contains the microfluidic chip and the optical sensing components, holds the channel aligned with the LED and the two LDRs, and encloses the sensing area to reduce the ambient light reaching the sensors.

The system operated under a water-in-oil droplet configuration. Deionized (DI) water was used as the dispersed phase, while paraffin oil (Fluka Analytical, Sigma-Aldrich, Steinheim, Germany) served as the continuous phase, chosen for its low viscosity and optical transparency. Fluid injection was performed using two identical syringe pumps (NE-1000, New Era Pump Systems Inc., Farmingdale, NY, USA) with 5 mL disposable syringes connected via Tygon tubing (0.4 mm inner diameter) to the inlets and outlets. The physical properties of both continuous and dispersed phases used in the experiments are summarized in Table 1.

Six independent experiments were performed to evaluate the performance of the sensor and to examine the influence of droplet velocity on monodispersity for DI-water emulsification in paraffin oil. Each experiment was conducted at a fixed Capillary number (Ca) of 0.006 and surface tension (σ) of 0.05 N/m, while varying the flow-rate ratio ($\phi =Q_{c}/Q_{d}$). A fixed continuous-phase flow rate ($Q_{c}$) was maintained, and the dispersed-phase flow rate ($Q_{d}$) was adjusted for each ϕ value, as summarized in Table 2. The ϕ values ($\phi_{Min}=0.5,\phi_{Max}=2.0$) selected ensured droplet formation within the dripping or squeezing regimes [11].

### Calibration Process

A calibration procedure was applied to each experimental condition to validate the velocity measurements obtained from the embedded sensor against an independent optical reference. Droplet motion was recorded at 60 fps using a microscope, and reference velocities were extracted using Fiji (version 2.16.0/1.54p) software [25] by analyzing contrast-enhanced grayscale recordings. The Z-profile intensity signals from two regions of interest (ROIs) separated by a known distance were cross-correlated to estimate the inter-frame lag, and parabolic interpolation was applied to the correlation peak to achieve sub-frame temporal resolution. This reduces the quantization error inherent to integer-frame detection (±0.5 frames, equivalent to ±6.25% at typical lag values) to ±0.1 frames (±1.25%).

Spatial calibration was performed using the fabricated channel width (1400 μm) as the reference standard, with uncertainty estimated at ±7 μm (±0.5%) based on the LPKF ProtoMat S63 milling machine specifications (positioning repeatability: ±1 μm) plus conservative margins for tool wear and thermal effects. Combined with pixel measurement uncertainty (±1.5%) in quadrature (±1.6%), the total spatial uncertainty is conservatively rounded to ±2% to account for additional unquantified sources such as edge-detection ambiguity and optical distortion at the channel boundaries. The reference velocity uncertainty was calculated using standard error propagation: $\delta V/V=\sqrt{{\left(\delta \Delta x/\Delta x\right)}^{2}+{\left(\delta \Delta t/\Delta t\right)}^{2}}$, yielding a combined reference uncertainty of ±2.4%. This methodology provides sufficient temporal and spatial resolution to accurately characterize droplet velocities in the 3–8 mm/s range investigated in this study.

To substantiate the agreement between the embedded sensor and the optical reference, Table 3 presents a comprehensive uncertainty budget that decomposes the velocity measurement uncertainty into its individual contributions for both methods.

The embedded sensor velocity is computed as V=d/Δt (Equation (1)), where d=9.88 mm is the center-to-center sensor spacing and Δt is the measured transit time. Applying standard error propagation, $\delta V/V=\sqrt{{\left(\delta d/d\right)}^{2}+{\left(\delta \Delta t/\Delta t\right)}^{2}}$. The distance uncertainty (±50 μm, based on the LPKF ProtoMat S63 milling tolerance for the PCB sensor footprint) contributes ±0.5%. The timing uncertainty of 653 μs—comprising the 500 μs microcontroller resolution and the 419 μs two-sensor LDR jitter, combined in quadrature—contributes less than 0.06% at the fastest tested velocity (8 mm/s, transit time 1235 ms). In absolute terms, the timing-limited velocity resolution $\delta V=V^{2}\;\delta \left(\Delta t\right)/d$ evaluates to 0.004 mm/s (0.05%) at 8 mm/s and 0.001 mm/s (0.025%) at 3.7 mm/s—both negligible compared to the dominant distance uncertainty. The combined sensor uncertainty is therefore dominated by the distance term at ±0.5%.

## 5. Results and Discussion

The embedded sensor was validated across six flow-rate ratios (ϕ=0.5–2.0), measuring droplet velocities from 3.7 to 7.4 mm/s with an average deviation of 5.32% from the optical reference. All results were obtained in a single autonomous run using the same materials, flow-focusing chip (1.4 mm channel width), and experimental setup, varying only the dispersed-phase flow rate ($Q_{d}$). The following subsections first validate the velocity measurement against the optical reference and then examine the relationships between velocity, droplet length, and monodispersity.

### 5.1. Validation of Embedded Droplet Velocity Measurement

**Figure 5:**
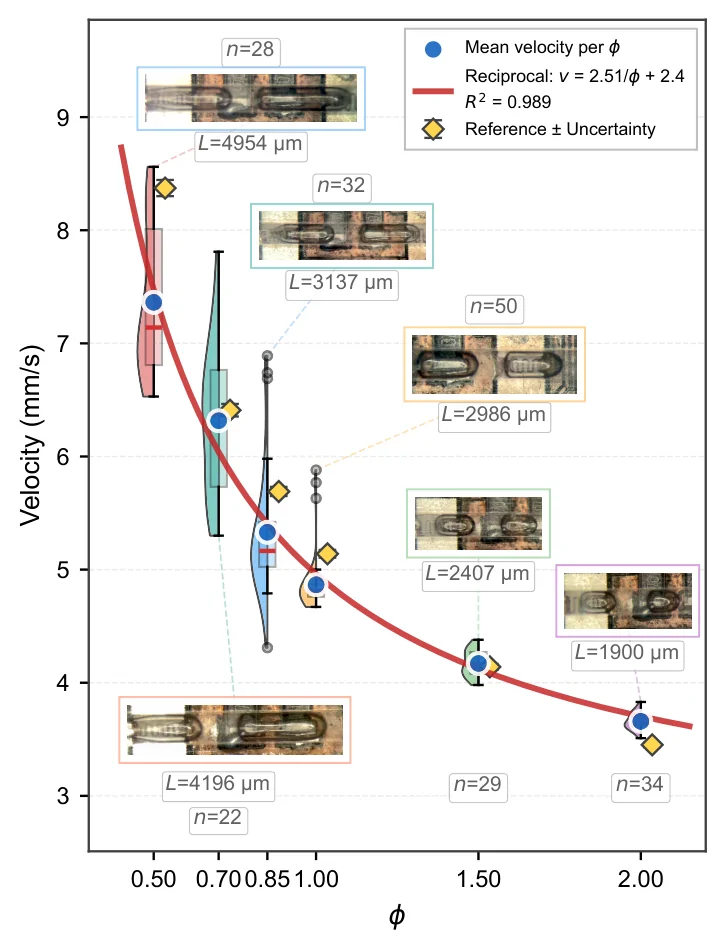
Droplet velocity distributions measured by the embedded sensor for each flow-rate ratio (ϕ), shown as violin plots with embedded box plots. Blue circular markers indicate the mean velocity per ϕ, while yellow diamond markers represent the optical reference values with associated uncertainties. Sample sizes (*n*) are displayed below each distribution. The dashed curve shows a reciprocal fit V=2.51/ϕ+2.45 ($R^{2}=0.989$). Embedded images show representative droplet formation with corresponding mean droplet lengths (*L*) for each ϕ.

Figure 5 presents the droplet velocity distributions measured by the embedded sensor for each flow-rate ratio (ϕ), together with the optical reference values and their associated uncertainties. As ϕ increased from 0.5 to 2.0, the mean droplet velocity decreased from approximately 7.4 mm/s to 3.7 mm/s, consistent with the reciprocal scaling predicted by flow conservation in single-regime flow-focusing operation. This trend is visually confirmed by the embedded images in Figure 5, which illustrate the transition from larger, faster droplets at low ϕ to smaller, slower droplets at higher ϕ. The droplet generation frequency, determined from the video recordings, remained between 1.05 and 1.17 Hz in all six conditions while $Q_{d}$ decreased fourfold, so the smaller and slower droplets at higher ϕ were produced at a nearly constant rate. For each condition, between 22 and 50 droplets were analyzed, yielding a validation subset of 195 droplets across all six conditions.

Spearman rank correlation computed on the mean velocities (n=6) yielded ρ=−1.0 (p<0.005), indicating a strictly monotonic ordering across the tested ϕ conditions. Given that the channel width (1.4 mm), continuous-phase flow rate ($Q_{c}$), and capillary number remained constant throughout all experiments, the system operated within a single hydrodynamic regime. Under these conditions, the total volumetric flow rate is given by $Q_{\mathrm{total}}=Q_{c}\left(1+1/\phi \right)$, and since the channel cross-section is fixed, the mean velocity is expected to follow V=b/ϕ+a, where *a* and *b* are constants determined by $Q_{c}$ and the channel geometry.

Fitting this reciprocal model to the measured data yielded V=2.51/ϕ+2.45 ($R^{2}=0.989$, p<0.001), reducing the unexplained variance by 93% relative to a linear fit ($R^{2}=0.85$, p=0.009). The monotonic response and reciprocal scaling confirm that the sensor accurately resolves deterministic changes in droplet transport dynamics imposed by the controlled nondimensional parameters.

The sensor readings show agreement with the optical reference (yellow markers), exhibiting an average deviation of 5.32% across all tested conditions, with per-condition deviations ranging from 0.74% (ϕ=1.50) to 12.07% (ϕ=0.50). The largest deviation was observed at ϕ=0.50, the condition that also exhibited the highest velocity dispersion among all tested ratios (see Figure 5), suggesting that increased droplet-to-droplet variability at this flow-rate ratio is the primary contributor to the elevated measurement error. Excluding this condition, the remaining five deviations remained below 6.4%, confirming the overall reliability of the embedded detection method based on Equation (1). As detailed in the uncertainty budget (Table 3), the combined measurement uncertainty of ±2.4% (dominated by the optical reference) accounts for the majority of the observed deviations, with the residual discrepancy likely attributable to droplet-to-droplet velocity variability within each experimental condition.

### 5.2. Velocity and Monodispersity Analysis

**Figure 6:**
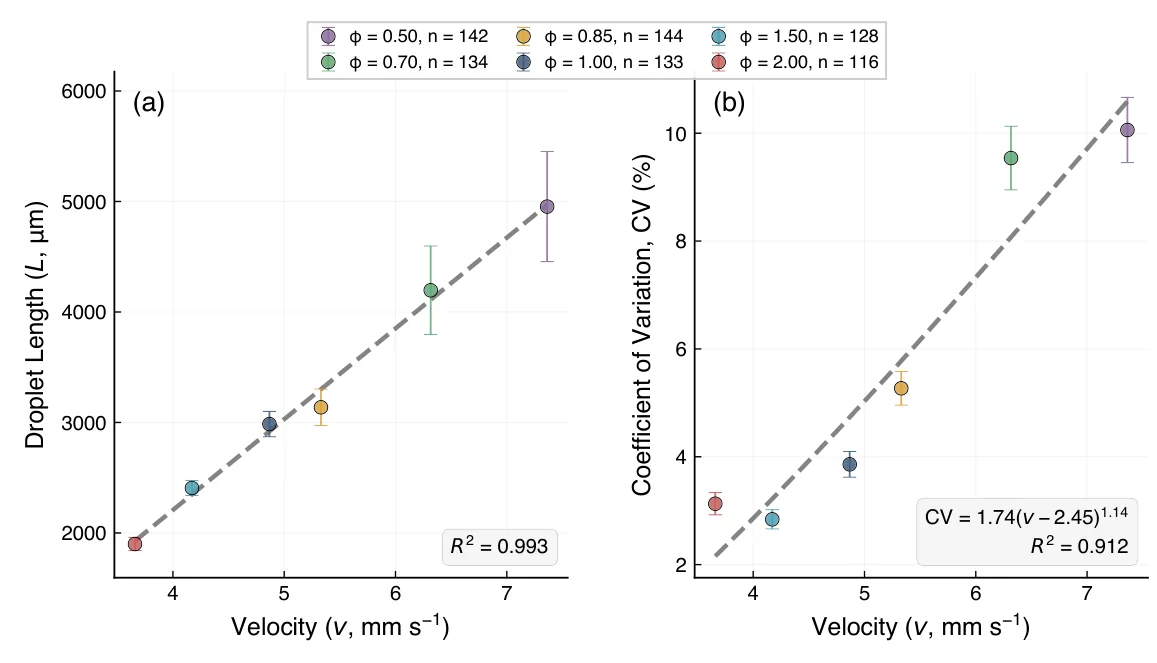
Relationship between mean droplet velocity and (**a**) droplet length and (**b**) coefficient of variation (CV) for different flow-rate ratios (ϕ). Each data point represents the mean ± uncertainty (*n* shown per condition). A linear fit ($R^{2}=0.993$; n=6) is shown in (**a**), while the physically constrained power-law model of eq:cvmodel ($R^{2}=0.912$; n=6) is shown in (**b**).

To investigate the relationship between droplet velocity and monodispersity, the system first determined individual droplet lengths and then computed the corresponding coefficient of variation (CV) for each operating condition, as summarized in Figure 6.

Figure 6a presents the mean droplet length measured by the embedded sensor as a function of velocity for each flow-rate ratio (ϕ). Consistent with the results in Figure 5, as ϕ increased, both droplet velocity and droplet length decreased, revealing a positive linear relationship between these two parameters. As the velocity increased from 3.7 mm/s to 7.4 mm/s, the mean droplet length rose from approximately 1900 μm to 4950 μm. For each condition, between 116 and 144 droplets were measured by the sensor, yielding a total of 797 droplets analyzed across all tests. Additionally, Figure 6a includes inset images corresponding to each mean droplet-length value, providing direct optical visualization of the increase in droplet size with velocity. Although based on a limited number of operating conditions (n=6), the relationship between droplet velocity and mean length exhibited a positive linear trend, supported by both parametric (Pearson r=0.997, p<0.001) and non-parametric (Spearman ρ=1.0, p<0.005) analyses. Linear regression yielded a slope of 821 μm·s/mm (95% CI: [728, 915]), with $R^{2}=0.993$. This trend is consistent with the expected dependence of droplet size on flow dynamics in flow-focusing systems. A post hoc power analysis for the length–velocity correlation (r=0.997, n=6, α=0.05, two-tailed) yielded achieved power exceeding 0.99, well above the conventional 0.80 threshold; however, post hoc power is conditioned on the observed effect size, and the small sample size limits the generalizability of the precise regression parameters.

Figure 6b presents the variation in monodispersity, expressed as CV, as a function of droplet velocity. Although a simple linear regression describes the measured data within the experimental range, such a model predicts non-physical negative variability outside the domain of observation. To ensure physical admissibility (CV≥0) and maintain consistency with droplet formation scaling, a power-law formulation anchored at the asymptotic velocity term was adopted.

Starting from the experimentally validated relationships L/W=1+α/ϕ and V=a+b/ϕ, elimination of ϕ yields a linear dependence of *L* on (V−a). Furthermore, the experimentally supported scaling $\sigma_{L}\propto L^{\gamma }$ implies

(3) $$\mathrm{CV}=\frac{\sigma_{L}}{L}\propto L^{\gamma -1}\propto {\left(V-a\right)}^{k},$$

where k=γ−1. The resulting model is expressed as

(4) $$\mathrm{CV}=C{\left(V-a\right)}^{k}.$$

Nonlinear least-squares regression of Equation (4) yielded C=1.74 and k=1.14, with $R^{2}=0.912$. The model is defined for V≥a, where *a* represents the asymptotic minimum velocity obtained from the reciprocal flow-rate fit. As all measured operating points satisfy V>a, the formulation inherently enforces non-negative variability and provides a physically admissible description of velocity-dependent droplet monodispersity within the investigated ϕ range of 0.5–2.0 for water-in-paraffin-oil emulsions in a 1.4 mm PMMA flow-focusing channel.

As an internal consistency check, the assumption $\sigma_{L}=\beta \;L^{\gamma }$ underlying Equation (3) was validated independently via log-log regression, yielding γ=2.47 (95% CI: [1.71, 3.22]; $R^{2}=0.954$, p<0.001). The exponent derived from the CV–velocity fit (k=1.14, i.e., γ=k+1=2.14) falls within this confidence interval, supporting the physical basis of the model. Both regressions are based on n=6 operating conditions, and the parameters should be regarded as preliminary estimates specific to the tested configuration.

The results presented above should be interpreted in light of the experimental scope. All measurements were obtained with a single fabricated PCB sensing module, a single channel geometry (1.4 mm width, square cross-section), a single fluid system (water-in-paraffin oil), and a fixed capillary number (Ca=0.006), which limits direct generalization to other configurations. The sensor spacing of 9.88 mm, while suitable for the 3–8 mm/s velocity range tested here, would require adaptation for significantly slower or faster droplet regimes. At the upper end, the maximum detectable velocity is set by the LDR detection delay and increases linearly with droplet length, as established by Equation (2) and detailed in Section 2. Temperature-dependent drift of the LDR resistance was not characterized; however, all experiments were conducted at ambient laboratory conditions (20–25 °C) within a single session, minimizing thermal effects. Because a single LED illuminates both LDRs and the velocity is obtained from the time difference between the S1 and S2 transitions, fluctuations in LED intensity or ambient temperature act on both sensors alike and largely cancel in Δt, leaving only the effect of differences between the two sensors. A drift large enough to bring the baseline of one sensor close to its comparator reference would affect detection rather than timing, and is compensated by re-adjusting the digital-potentiometer reference of that sensor against its baseline.

The empirical correlations are also specific to the tested conditions. The linear length–velocity relationship derives from the Garstecki squeezing-regime scaling (L/W=1+α/ϕ) [19], where *W* enters directly; different channel widths would alter the regression parameters. At higher capillary numbers, achievable through lower interfacial tension, higher viscosity, or increased flow rates, the system would transition to the dripping regime, fundamentally changing the length–velocity dependence [20]. The CV–velocity power-law parameters (*C*, *k*) in eq:cvmodel are similarly regime-specific, as the viscosity ratio and interfacial tension affect both droplet size variability and the transition between formation regimes. While the sensor hardware measures transit time independently of the formation mechanism, sensor spacing and comparator thresholds may require adaptation for sub-millimeter channels or fluid pairs with reduced refractive-index contrast. Future work should therefore re-establish these correlations across multiple channel geometries (e.g., T-junction), fluid systems, and capillary numbers.

## 6. Conclusions

This work presented the experimental validation of an embedded velocity sensing system for droplet-based microfluidics. The sensor integrates LDR-based optoelectronic detection and interrupt-driven signal processing within a compact, low-cost platform designed for integration into Lab-on-PCB devices. Its architecture was developed using a Model-Based Systems Engineering approach in Ansys SCADE, which facilitated modular integration, simulation-based verification of the embedded logic, and C code generation.

The maximum detectable velocity, set by the LDR detection delay ($t_{\mathrm{detect}}\approx 11.5$ ms), increases linearly with droplet length and is approximately 522 mm/s for 6 mm plugs. The sensor was validated using a 1.4 mm flow-focusing geometry with flow-rate ratios ϕ=0.5–2.0, measuring 22–50 droplets per condition for a total of 195 droplets tested. The average velocity deviation from optical reference measurements was 5.32%, with per-condition deviations ranging from 0.74% to 12.07%. The largest deviation was observed at ϕ=0.50, the condition exhibiting the highest velocity dispersion.

An embedded analysis across 797 droplets (116–144 per condition) revealed a positive linear trend between mean droplet length and velocity ($R^{2}=0.993$). Additionally, the relationship between velocity and monodispersity followed the physically constrained power-law model $\mathrm{CV}=C\;{\left(V-a\right)}^{k}$ ($R^{2}=0.912$). This formulation is grounded in the Garstecki squeezing-regime scaling and flow conservation principles, combined with an experimentally validated assumption on length variability. It captures the observation that larger, faster droplets exhibit disproportionately greater size variability, indicating reduced monodispersity at higher velocities, and inherently ensures non-negative predictions across the operating range. Both trends are based on n=6 operating conditions and are specific to the tested configuration.

In summary, these results demonstrate the effectiveness of the proposed system in enabling autonomous, real-time characterization of droplet formation dynamics within flow-focusing devices. Future work includes evaluation across alternative droplet generators (e.g., T-junction, co-flow) and sub-millimeter geometries, different fluid systems and higher capillary numbers, a denser set of flow-rate ratios to refine the empirical correlations, assessment of reproducibility across independently fabricated sensor modules, integration of closed-loop flow-rate control, and demonstration in targeted applications such as nanoparticle synthesis and drug encapsulation monitoring.

## Figures and Tables

**Table 1:** Fluid Properties Used in the Experimental Setup.

Phase	Fluid	Property	Value	Units
Continuous	Paraffin oil	Dynamic viscosity (η)	145	mPa·s
		Density (ρ)	∼858	kg/$m^{3}$
Dispersed	DI Water	Dynamic viscosity (η)	1.0	mPa·s
		Density (ρ)	998	kg/$m^{3}$

Note: All fluid properties were measured or referenced at room temperature (20–25 °C).

**Table 2:** Flow-Rate Ratios and Operating Conditions Used in the Experiments.

Experiment	ϕ	$Q_{c}$ (mL/h)	$Q_{d}$ (mL/h)
1	0.50	14.59	29.19
2	0.70	14.59	20.86
3	0.85	14.59	17.17
4	1.00	14.59	14.59
5	1.50	14.59	9.732
6	2.00	14.59	7.299

Note: $Q_{c}$ and $Q_{d}$ correspond to the continuous- and dispersed-phase flow rates, respectively, used to define the flow-rate ratio $\phi =Q_{c}/Q_{d}$.

**Table 3:** Velocity Measurement Uncertainty Budget.

Source	Description	Contribution
Optical Reference Method		
Temporal	Sub-frame interpolation	±1.25%
Spatial	Channel width calibration	±2.0%
Subtotal	(quadrature sum)	±2.4%
Embedded Sensor		
Distance (*d*)	PCB manufacturing (9.88 mm)	±0.5%
Timing (Δt)	MCU resolution + LDR jitter	<0.06%
Subtotal	(quadrature sum)	±0.5%
Combined Comparison		
Sensor vs. Reference	Root-sum-square of both	±2.4%

Note: Timing contribution computed at the fastest expected velocity (8 mm/s, Δt=1235 ms). At lower velocities, the timing contribution decreases further.

## Data Availability

The datasets generated and analysed in this study are openly available in IEEE DataPort at https://doi.org/10.21227/tgm0-dr17.

## Abbreviations

The following abbreviations are used in this manuscript:

ADC	Analog-to-digital converter
CPS	Cyber–Physical System
CV	Coefficient of variation
DI	Deionized (water)
ISR	Interrupt Service Routine
LDR	Light-dependent resistor
LED	Light-emitting diode
LoC	Lab-on-Chip
LoPCB	Lab-on-PCB
MBD	Model-Based Design
MBSE	Model-Based Systems Engineering
MCU	Microcontroller unit
PCB	Printed circuit board
PCR	Polymerase chain reaction
PMMA	Polymethyl methacrylate
ROI	Region of interest
